# Supplementary material for: Impact of Visual Cues on Consumers’ Freshness Perception of Prepared Vegetables
Source: Foods. 2024 Oct 21;13(20):3342. doi: 10.3390/foods13203342 (PMC11507573; doi:10.3390/foods13203342)
Supplement: Supplementary file 1 [file foods-13-03342-s001.zip › foods-3172588-supplementary.pdf]

Supplementary data

Table S1: ANOVA model fitted using factors “Product” (Fixed – 27 levels) and “Assessor” (Random – 122 levels)

	Df	Sum Sq	Mean Sq	F-value	p-value
Factor (Assessor)	121	2375.48	19.63	16.65	<0.001
Factor (Product)	26	1130.82	43.49	36.89	<0.001
Residuals	3146	3709.18	1.18		

Table S2: ANOVA model fitted using data from the 21 samples listed in Table 2 and using factors “Shape“ and “Presence of vegetable (Yes/No)”

	Df	Sum Sq	Mean Sq	F-value	p-value
Factor (Shape)	2	4.51	2.25	24.19	<0.001
Factor (Carrot)	1	0.28	0.28	2.99	0.10
Factor (Beetroot)	1	1.35	1.35	14.43	<0.01
Factor (Green pepper)	1	0.25	0.25	2.69	0.12
Residuals	15	1.40	0.09		

Table S3: ANOVA model fitted using data from the 21 samples listed in Table 2 and using factors “Shape“ and “Number of vegetables”.

	Df	Sum Sq	Mean Sq	F value	p-value
Factor (Shape)	2	4.51	2.25	11.35	0.001
Factor (Number of vegetables)	2	0.09	0.05	0.24	0.79
Residuals	16	3.18	0.20		
